# Online Discussions About Alopecia Areata in 5 Arabian Gulf Countries: Social Media Listening Study

**DOI:** 10.2196/78262

**Published:** 2026-08-13

**Authors:** Ashraf Reda, Ghadear AL Alawi, Ameen Al Awadhi, Amina Al Obaidli, Fadwa Ebeid, Haytham Mohamed Ahmed

**Affiliations:** 1 Department of Dermatology, Mediclinic Welcare Hospital, Dubai, United Arab Emirates; 2 Department of Dermatology, Adan hospital, Hadiya, Kuwait; 3 Department of Dermatology, Salmaniya Medical Complex, Manama, Bahrain; 4 Department of Dermatology, Rumaila hospital, Doha, Qatar; 5 Department of Medical Affairs, Pfizer Inc Ltd, Building 6, Dubai Media City, Dubai, United Arab Emirates, 971 501656046

**Keywords:** alopecia areata, social media, patient concerns, search patterns, online discussions, search trends

## Abstract

**Background:**

Alopecia areata (AA) is an autoimmune disease that can affect the quality of life of affected individuals. Publicly available online conversations and search behavior provide valuable insights into public awareness, understanding, and information related to AA. An infodemiology approach, therefore, offers an opportunity to characterize these perspectives across Gulf countries. This study evaluated the understanding and awareness of AA in Bahrain, Kuwait, Oman, Qatar, and the United Arab Emirates based on AA-related mentions and internet searches.

**Objective:**

Despite its prevalence, AA is often misunderstood, particularly in the Middle East. By examining the data from social media platforms, online forums, and internet searches, this study provided insight into the regional AA perceptions and awareness in these 5 Gulf countries.

**Methods:**

Online discussions in English and Arabic mentioning AA and related terms were analyzed from the 5 countries between January 2022 and April 2023.

**Results:**

The analysis of more than 8500 mentions of AA over 16 months revealed significant online engagement, especially from the United Arab Emirates. Educational content dominated the discussions, with a notable increase following the Oscars incident in April 2022. Gulf news platforms and X (formerly Twitter) were the primary discussion platforms, with health care professionals mainly using English and patients using both English and Arabic. Common topics included potential contributors to AA, emotional distress, and the AA patient journey.

**Conclusions:**

The study identified gaps in awareness and understanding of AA among patients, health care professionals, and medical facilities in the Gulf region through online discussions and search patterns. These insights can facilitate the development of targeted educational initiatives, support strategies, improve awareness, reduce stigma, and enhance support networks for those affected by AA.

## Introduction

Alopecia areata (AA) is an autoimmune disease that results in nonscarring hair loss, commonly manifesting as well-defined patches on the scalp or other body areas [1]. It can occur at any age and affects both men and women, leading to significant psychosocial distress due to its visible effect on the physical appearance. AA affects approximately 2% of the general population at some stage in their lives, as evidenced by numerous epidemiological studies conducted across Europe, North America, and Asia [1]. In the Middle East, Saudi Arabia reported prevalence rates between 2.3% and 13.8%, while in Africa, prevalence rates were lower, such as 0.1% in Nigeria and 0.2% in South Africa [2]. These variations can be attributed to differences in genetic, environmental, and health care factors.

A study conducted in 2021 showed that Facebook, Twitter, Instagram, TikTok, YouTube, Snapchat, and other social media platforms help users share dermatology-related content, seek medical advice, share photos and videos, and build social networks by interacting with other users, thereby benefiting greatly from the platforms’ visual nature [3]. Another study showed that a significant number of participants with dermatological conditions (82.4%) used the internet for information, and 65.4% specifically used social media [4]. AA is a frequently searched term on Instagram, indicating high engagement among those affected [5]. By analyzing online discussions and search trends related to AA, researchers and health care professionals (HCPs) can gain insights into regional perceptions, common beliefs, challenges, and levels of awareness. A 2019 study reported that social media can help develop instruction for dermatology trainees, facilitate new research methods, and foster patient-centered perspectives and advocacy groups [6]. Using online data analytics, key themes and patterns in AA-related interactions can be identified, aiding the development of public health campaigns and educational initiatives, enhancing awareness, reducing stigma, and improving support for those affected by AA.

Infodemiology refers to the study of the distribution and determinants of information shared through electronic media, particularly the internet, with the aim of informing public health strategies and policy decisions [7]. Social media analysis provides large-scale, real-time data that allows researchers to explore human behavior, track emerging trends, and understand public perceptions [8]. Unlike traditional surveys or focus groups, which are limited by sample size and response bias [9], using data from social media to capture the real-world patient journey, alongside traditional epidemiological studies, sheds light on unmet needs, improves patient care pathways, develops tailored treatment pathways, and helps future researchers address the complexities of AA management [10]. Online discussions often illuminate dimensions that may be underrepresented in clinical datasets, including experiences of stigma, emotional distress, identity challenges, and social burden. By capturing these experiences in real time, social media analysis complements conventional research methods and contributes to a more holistic framework for addressing the complexities of AA management. The study was grounded in infodemiology, and the findings informed the study design and research questions, justifying a focus on real-world online discourse and platform-based differences in AA communication. The framework further guided the interpretation of results by contextualizing observed patterns within established evidence on patient information-seeking behavior and the variability of online health content.

## Methods

### Study Design

The AA social analytics study was commissioned by Pfizer in April 2023 and conducted by Rila Global Consulting. The company scanned more than 100 million websites, analyzing up to 16 months of data from January 2022 to April 2023. This research focused on the United Arab Emirates, Kuwait, Bahrain, Oman, and Qatar and included content in both English and Arabic.

### Data Sources and Collection

Rila Global Consulting crafted specific queries to retrieve mentions of AA-related terms, abbreviations, and hashtags associated with AA. They conducted searches for related products and identified key influencers by monitoring online engagement metrics. Authors with significant impact or visibility were selected as top influencers. The system scanned publicly available data on social media sites such as X (formerly Twitter), blogs, online forums, news outlets, and health information websites. Content originating from the United Arab Emirates, Kuwait, Bahrain, Oman, and Qatar was included in the analysis. Both English- and Arabic-language content was captured. All data were obtained exclusively from publicly accessible sources. No private accounts, password-protected content, or personally identifiable information were accessed or retained.

### Search Strategy and Query Development

A structured query library was developed to retrieve AA-related content with high specificity. The query set included medical terminology related to alopecia areata, common abbreviations such as AA, lay expressions, and colloquial terms related to hair loss, hashtags associated with AA discussions, and product-related keywords associated with management, supplements, cosmetic solutions, and more. Queries were refined to minimize irrelevant results, particularly where “AA” appeared as an unrelated abbreviation; therefore, the dataset reflected genuine AA-related discourse.

### Data Cleaning and Filtering

After data extraction, the dataset underwent a multistage cleaning process. This included the removal of duplicate posts and syndicated content to prevent repeated material. Language filtering was applied to retain only English- and Arabic-language content. Geolocation filtering was conducted using available metadata and platform-specific location indicators to ensure relevance to the targeted Gulf Cooperation Council countries. Bot-generated, spam, and automated content were excluded using algorithmic detection methods. The dataset was restricted to publicly accessible posts to maintain ethical compliance. No personal or identifying information was collected, stored, or analyzed at any stage of the study.

### Content Classification and Analysis

Content processing and classification were performed using vendor-implemented automation, including language identification, keyword and query matching, and rule-based parsing rather than trainable machine learning models. No supervised or unsupervised statistical natural language processing models (topic modeling, embeddings, or large language models) were trained or evaluated as part of this study. Posts were categorized across multiple dimensions, including topic (symptoms, diagnosis, emotional impact, and treatment discussions), sentiment (positive, negative, or neutral), audience type (patients, caregivers, HCPs, influencers, or the public), and platform characteristics (X, forums, or news websites). Automated outputs were supplemented by expert review to support contextual interpretation, particularly for Arabic-language posts and culturally specific expressions. Manual review was conducted to contextualize ambiguous posts, particularly in Arabic, but was not designed as a formal annotation exercise with a predefined sampling frame or dual independent coding. Accordingly, interrater agreement metrics were not calculated, and the analysis should be interpreted as descriptive rather than as a validated classification model.

### Analytic Approach and Tooling

The classification approach was explicitly described as rule-based, using vendor-implemented parsing, keyword and query matching, and language identification. No supervised or unsupervised machine learning model training was conducted in this study. The term “hybrid approach” was defined as a combination of automated, rule-based processing and manual contextual review, particularly for Arabic-language content.

### Bilingual Data Processing

English- and Arabic-language content was processed within a unified analytic workflow. Language identification was applied during data cleaning, and posts were classified using a shared rule-based framework across both languages without requiring full translation into a single language. Topic categories and labels were aligned through consistent keyword mapping and conceptual equivalence of medical and colloquial terms related to alopecia and hair loss. Expert manual review was conducted to support contextual interpretation, particularly for Arabic-language content. This review addressed dialectal variation, idiomatic expressions, and ambiguities that could not be fully resolved through automated rule-based processing. Although efforts were made to ensure consistency across languages, differences in linguistic structure and expression may have led to variability in classification.

### Influencer Identification

Influential authors were identified using a combination of quantitative and qualitative indicators. These included the frequency of AA-related posts, engagement metrics (likes, comments, shares, and reposts), follower count and overall visibility, cross-platform presence, and the relevance and consistency of AA-related content. Authors demonstrating high reach, sustained engagement, or impact on online discussions were classified as key influencers.

### Quantitative Metrics

Standardized social analytics metrics were applied to quantify the scale and dynamics of AA-related conversations. These metrics included mentions, impressions, reach, engagement, traffic, and follower-based indicators, as defined in Table 1. The metrics were used to compare patterns across countries and digital platforms and to identify trends over the study period.

**Table 1.** Key terms and metrics on alopecia areata study in Gulf countries.

Term	Definition
Mention	Mentioning a brand, a product, or an account in an online post
Author	The nickname, username, or full name of the individual or organization who posted a mention
Tweets (post)	A post on X (formerly Twitter)
Retweets (repost)	Resharing another account’s post to all followers by clicking on the repost (retweet) button
Following	The number of users the author follows
Followers	The number of users following the author
Impressions	The combined sum of the followers of the original author and the followers of any user who reshared the post
Reach	An estimate of the number of individuals who may have seen a piece of content based on engagement (eg, likes, comments, retweets, and replies) and traffic (eg, average site visitors and monthly visitors), allowing comparison across data sources and platforms
Engagement	The total number of times a user interacts with a tweet or post, as measured by a range of metrics that include likes, comments, replies, shares or retweets, click-throughs, and saves
Traffic	The average number of visitors to a website, mobile app, or profile

### Descriptive Nature of Analysis

The analytical approach used in this study was both descriptive and exploratory. Data were gathered from publicly available online discussions via query-based retrieval rather than structured sampling methods. As a result, the findings reflect trends in digital discourse and are not meant to represent population-level estimates or support inferential statistical conclusions.

### Ethical Considerations

This study analyzed publicly available data from online platforms and social media sources in English and Arabic. No private, restricted, or password-protected content was accessed. No personal identifiers were collected, stored, or reported. All data were analyzed in aggregate form, and individual users were not identifiable. The study adhered to established ethical principles for internet-mediated research and complied with applicable data protection standards. As the research involved the analysis of publicly available content and did not include human participants or intervention, formal ethics committee approval was not required.

## Results

### Overview

The analysis of online discussions about AA revealed several prevalent themes, misconceptions, and regional differences. Key topics included AA discussions in the Gulf, contributors to hair loss, treatment options, experiences with AA, emotional impacts, and coping strategies. Search trend analysis demonstrated differences in query volume and topic patterns across Gulf countries. English was predominantly used by HCPs and medical institutions, whereas patients used a combination of Arabic and English. Interchangeable use of terms such as “baldness,” “hair loss,” and “alopecia” was observed across discussions.

### Impact of the Oscars Incident on AA Discourse in the Gulf Region

**Figure 1.**
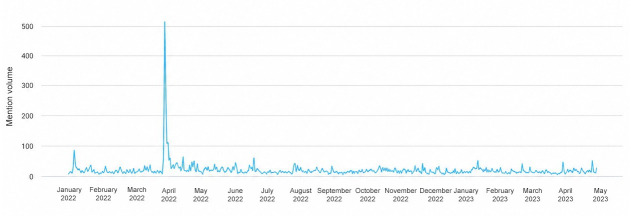
Monthly volume of alopecia areata mentions in the Gulf region, showing a marked spike in April 2022.

The volume of discussions on AA in the Gulf region remained relatively stable month over month, with a marked spike occurring during the Oscars in 2022, as shown in Figure 1. During the same period, alopecia was referenced in widely circulated media coverage [11]. However, this observation reflected a temporal association and should be interpreted as hypothesis generating rather than indicative of a causal relationship.

### Content Source Analysis: AA Discussions in the Gulf (2022-2023)

Between 2022 and 2023, discussions on AA in the Gulf were primarily concentrated on news platforms and X (formerly Twitter). News sources accounted for 4505 (53%) of total mentions (N=8500), followed by X at 3655 (43%), and Tumblr at 340 (4%).

### Gulf Region Country Analysis: AA Discussions

Discussions on AA in the Gulf region predominantly originated from the United Arab Emirates, which accounted for 5695 (67%) of total mentions (N=8500). Bahrain and Kuwait each accounted for 850 (10%) of the share of voice, followed by Qatar 680 (8%) and Oman 425 (5%).

### Social Media Analysis: Dominance of Educational Content in AA Discussions

Social media discussions on AA were predominantly educational or informational in nature, accounting for 4692 (55.2%) of analyzed content (N=8500). Personal stories comprised 2015 (23.7%), followed by HCP interactions (n=1071, 12.6%) and references to medical journals or research articles (n=722, 8.5%), as shown in Table 2.

**Table 2.** Content types shared online about alopecia areata (N=8500).

Category	Value, n (%)
Educational or informational	4692 (55.2)
Personal stories	2015 (23.7)
Health care professional interactions	1071 (12.6)
Medical journals and research articles	722 (8.5)

### Causes and Risk Factors

Posts included references to stress, dietary practices, hijab wearing, and environmental exposures as potential contributors to hair loss or alopecia. Users shared personal experiences and coping strategies. These observations reflect online discourse and are not intended to imply causal relationships.

Social media platforms are increasingly used by individuals experiencing hair loss as sources of medical information and patient education. However, much of the content available on these platforms promotes hair care products and treatments that lack scientific evidence [12]. HCPs use social media to educate patients about alopecia, clarify misconceptions about hair oils, and emphasize evidence-based treatments such as minoxidil, finasteride, and spironolactone pills. They also provide insights into the effects of the COVID-19 pandemic on hair health and discuss preventive measures against baldness, cautioning against hairstyles such as the “ponytail.” Furthermore, they share information about new treatments such as microscopic cell technology and emphasize the importance of consulting with physicians for tailored treatment plans, including options such as hair transplantation, Regenera injections, platelet-rich plasma (PRP) therapy, and mesotherapy.

Patients with alopecia typically seek advice from dermatologists and cosmetologists. Cosmetic surgeons were mentioned mainly in promotional contexts. Most educational content about alopecia from HCPs and medical facilities is in English, which may restrict access for Arabic-speaking individuals. Some facilities address this by offering multilingual resources.

**Figure 2.**
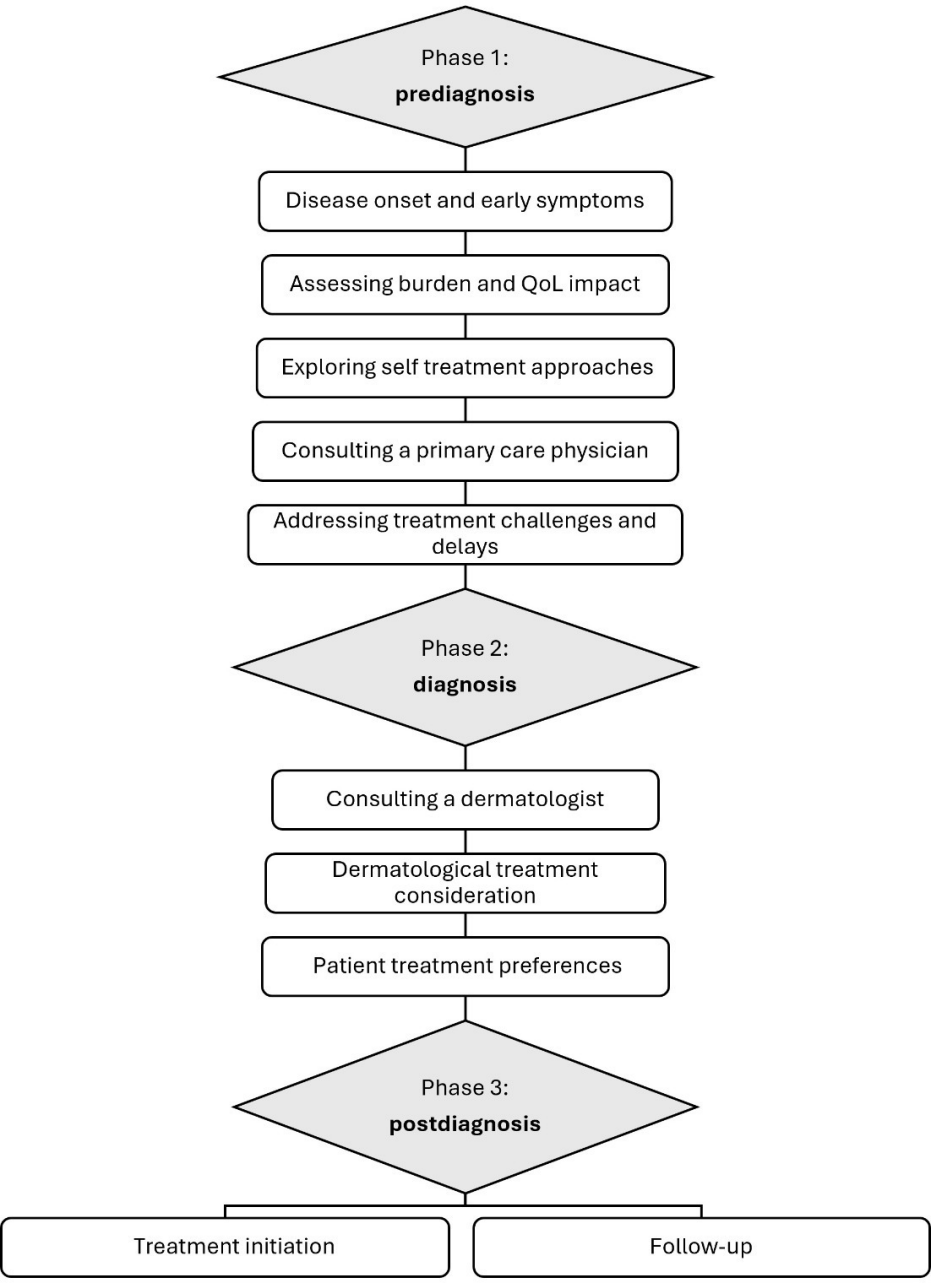
Alopecia areata patient journey during the prediagnosis, diagnosis, and postdiagnosis phases. QoL: quality of life.

Patients’ journeys in seeking solutions for alopecia vary in duration Figure 2, with many starting with medical treatments but eventually exploring natural products or alternative methods if conventional treatments prove ineffective [13]. Some patients may initially try medical treatments such as injections and creams provided by hospitals or dermatologists but may turn to alternative remedies based on recommendations from relatives or social circles if these treatments fail. There was a disparity in the use of clinical language when discussing alopecia compared to nonclinical language, indicating potential gaps in understanding and awareness of alopecia as a medical condition in the Gulf region and suggesting a need for improved education and awareness efforts.

Common treatments included topical corticosteroids, minoxidil, and intralesional corticosteroids, which help reduce inflammation, promote hair regrowth, and manage hair loss [14,15]. In the Gulf region, clinics frequently advertised hair transplant services using search terms such as “hair loss.” In contrast, despite the availability of PRP therapy, patient discussion regarding this modality appeared limited. Homeopathy was also promoted as a treatment option, alongside various products such as dietary supplements for hair loss, shampoos, and conditioners. Natural remedies and foods believed to aid hair regrowth, such as green tea, aloe vera, and garlic, are frequently discussed. Dermarollers were promoted for hair regrowth, with stress, pollution, and harsh chemicals identified as contributing factors to hair loss.

News about the approval of JAK (janus kinase) inhibitors for the treatment of alopecia was widely shared across the Gulf region, with foreign HCPs, local authors, and media outlets disseminating the information [16].

Authors in the Gulf region also shared news about a South Korean politician promising free baldness treatments in exchange for votes. The ruling party’s presidential candidate, Lee Jae-myung, proposed covering baldness treatment under public health insurance, which generated mixed reactions among voters [17]. This news sparked discussions among Gulf authors, highlighting the distinction between hair loss and alopecia. Prominent organizations, including dermatology societies across the Gulf, actively engaged in discussions on this topic, contributing to awareness and understanding in the region.

## Discussion

### Principal Results

Over a span of 16 months, from January 2022 to April 2023, we thoroughly analyzed approximately 8500 mentions of AA, with a significant 67% (5695) of these coming from the United Arab Emirates. Most content was educational (n=4692, 55.2%). The incident at the Oscars in April 2022 led to a regional spike in alopecia-related discussions. Users discussed AA across the region on platforms such as Gulf News (n=4505, 53%) and X (n=3655, 43%). English was predominantly used by HCPs, while patients used a combination of English and Arabic. Authors interchangeably used “baldness,” “hair loss,” and “alopecia” to enhance audience engagement. Fungal infections of the scalp, stress, diet, and environmental factors were discussed as leading contributors to alopecia. Many patients expressed emotional distress associated with alopecia. Natural remedies and self-treatment were frequently explored by patients, while beauty and medical centers advertised nonpharmacological treatments along with topical minoxidil.

### Causes and Risk Factors

In the Gulf region, discussions on hair loss and alopecia highlighted several factors: scalp fungal infections were mentioned in 1% of all conversations, while stress, dietary choices, hijab wearing, and environmental factors were recognized as possible contributors to hair loss.

#### Scalp Hair Fungal Infection

According to reports from news outlets, the German Association of Dermatologists has identified head fungus as one of the skin conditions likely leading to baldness or alopecia. Fungal infections are known to trigger hair loss, often accompanied by itching. Tinea capitis, a condition affecting both children and adults, manifests as scalp eruptions and hair loss [18]. Discussions on social media concerning both hair and head fungus and their effects on hair loss and alopecia constituted 1% of all posts analyzed.

#### Stress

Users engaged in discussions regarding the correlation between stress and hair loss, highlighting the importance of managing stress to reduce or reverse the effects of alopecia. Chronic stress leads to hair loss, although the exact mechanism is unknown [19]. Personal stories were shared by users on how stressful life events, such as school exams, divorce, or family illness, could exacerbate hair loss. Users expressed the challenging reality of living with alopecia, describing a vicious cycle where hair loss induces stress and emotional distress, thereby worsening and accelerating the condition.

#### Dietary Choices

Users actively discussed the influence of diet on hair loss, suggesting that crash diets and unhealthy eating habits can disrupt hormonal balance and lead to increased hair loss. Some users shared personal experiences of certain diets exacerbating hair loss, whereas others advocated for the benefits of maintaining a healthy and balanced diet to manage alopecia. Personal experiences were also shared, underlining how adopting nutritious eating habits had improved hair health and reduced hair loss for some individuals. Users offered tips and advice on specific foods and supplements that can be beneficial in managing alopecia. It is essential to address and rectify any nutritional deficiencies in patients experiencing hair loss [20].

#### Hijab Wearing

Users expressed concerns that wearing a hijab, a head covering worn by Muslim women, increases the rate of hair loss in individuals with alopecia. Some users proposed that the lack of exposure to vitamin D due to wearing a hijab could be a contributing factor to hair loss. This discussion sparked conversations about strategies to prevent or reduce hair loss in patients with alopecia who choose to wear a hijab. Conversely, other social media users recommended headscarves as a practical solution for covering bald patches caused by alopecia. They argued that headscarves can offer a way for individuals who are self-conscious about their hair loss to feel more comfortable and confident in public settings.

#### Environmental Factors

Discussions also frequently referenced environmental contributors, including pollution and water quality, as potential factors associated with hair loss. Users reported experiences suggesting that relocating to different cities influenced the severity of hair loss. Discussions surrounding alopecia and hair loss on social media platforms in the Gulf region may be relevant for pharmaceutical and aesthetic stakeholders seeking to understand public interest and awareness. Water filters were frequently mentioned among products discussed by companies, with associated keywords and hashtags appearing in online conversations about alopecia and hair loss.

### Comparison of Gulf Discourse Patterns With Global Studies on Dermatology-Related Social Media Content

The discourse patterns observed in the Gulf region align with broader international research on dermatology-related social media engagement. Several studies have demonstrated that patients with chronic skin conditions such as acne, vitiligo, and psoriasis use social media and online platforms to seek information, share experiences, and influence health care decisions [21-23]. Misinformation, terminology confusion, and commercial influence have also been identified as recurring challenges in digital dermatology conversations worldwide [24].

### Cultural Dimension of Alopecia Discourse in the Gulf

The cultural context of the Gulf region may influence how AA is discussed online. Religious, social, and gender norms may appear to shape disclosure behaviors, particularly among women, for whom hair functions as an important marker of social identity and personal meaning. The wearing of the hijab, for example, emerged both as a contributor to hair loss and as a coping strategy for concealing visible symptoms. A study investigated alopecia among Muslim women who self-reported hair loss related to wearing the hijab [25]. The study revealed that a significant number of Muslim women experienced alopecia, and they believed it is associated with hijab wear. More than one-third of the surveyed women were unaware of hijab-associated alopecia. The study discussed the importance of educating Muslim women on ways to mitigate the risks of alopecia or treat it without compromising their religious beliefs or values [25].

Language use also reflected cultural positioning, with Arabic more common in patient-driven discussions and English predominating in HCP communication. These findings suggest that online discourse around alopecia in the Gulf is embedded within broader sociocultural dynamics rather than being purely biomedical in nature.

### Limitations

One significant limitation of our study is the exclusion of data from Facebook and Instagram, as these platforms do not allow data crawling. This restriction clearly impacts the comprehensiveness of our analysis, particularly for social media platforms where data collection is limited. The analysis therefore reflects discussions from publicly accessible, primarily text-based platforms, potentially underrepresenting conversations occurring within private networks or visually driven platforms. The findings were based on a fixed 16-month observation period (2022‐2023). As social media dynamics evolve rapidly, the identified trends may not fully capture longer-term or ongoing shifts in discourse. Automated language processing and rule-based classification were used in the analysis of bilingual content. Although efforts were made to ensure accuracy, automated processing may introduce translation or categorization bias. The absence of full translation may have resulted in minor inconsistencies in cross-language classification.

Another limitation of this study is that the manual review conducted to contextualize ambiguous posts, especially those in Arabic, was not structured as a formal annotation exercise. It lacked a predefined sampling frame and did not involve dual independent coding. As a result, interrater agreement metrics were not calculated, meaning the findings should be viewed as descriptive rather than indicative of a validated classification model. This limitation may affect the reliability and generalizability of the analysis.

### Comparison With Prior Work

A literature review of AA in the Middle East and Africa discussed the prevalence, quality of life, and treatment modalities for AA [2].

A study by Gümüş [5] on social media use in patients with AA using questionnaires showed that 72.6% of the patients used social media to know about AA. The most commonly used platforms were Google (58.5%), Instagram (31.4%), and YouTube (30.5%). In contrast, in our study, users discussed AA across the region on platforms such as Gulf News (4505/8500, 53%) and X (3655/8500, 43%) [5].

An analysis of alopecia-related content on Instagram and TikTok examined popular posts within 10 hair loss–related hashtags on both platforms. Of the 90 posts analyzed within Instagram, nonmedical professional influencers were responsible for 66% of the posts, hair and wig companies for 29%, and medical professionals for only 4%. Of the 100 TikTok posts analyzed, influencers and patients each created 38%, while hair and wig companies contributed 14%, medical professionals accounted for 10%, and none of the top posts were created by board-certified dermatologists [12].

### Conclusions

The analysis of online discussions surrounding AA in the Gulf region provided insights into the perceptions and challenges associated with this condition. Throughout the study, we observed a variety of themes, including treatment options and emotional impacts. Key findings included a predominance of discussions originating from the United Arab Emirates. The lack of understanding among patients about AA versus general hair loss leads to delayed diagnosis and inadequate treatment. This study hypothesizes that cultural and societal influences, such as the Oscars incident in 2022, significantly shape perceptions and discussions about AA. Both natural remedies and medical interventions were frequently discussed, but the promotion of nonevidence-based treatments and the lack of standardized treatment protocols highlight the need for evidence-based guidance. The emotional toll of living with AA is significant, affecting an individual’s well-being beyond hair loss.

In summary, the study emphasizes the diverse landscape of AA discourse in the Gulf region, encompassing variations in awareness, resource access, and the emotional and social challenges faced by individuals affected by the condition. Moving forward, efforts must focus on enhancing education, promoting evidence-based treatments, and providing holistic support for individuals affected by AA.

## Abbreviations

AA: alopecia areata
HCP: health care professional
JAK: janus kinase
PRP: platelet-rich plasma
